# Enhancing photoacoustic imaging for lung diagnostics and BCI communication: simulation of cavity structures artifact generation and evaluation of noise reduction techniques

**DOI:** 10.3389/fbioe.2024.1452865

**Published:** 2024-09-10

**Authors:** Chengpeng Chai, Xi Yang, Xurong Gao, Junhui Shi, Xiaojun Wang, Hongfei Song, Yun-Hsuan Chen, Mohamad Sawan

**Affiliations:** ^1^ CenBRAIN Neurotech., School of Engineering, Westlake University, Hangzhou, Zhejiang, China; ^2^ Institute of Advanced Technology, Westlake Institute for Advanced Study, Hangzhou, Zhejiang, China; ^3^ Research Center for Humanoid Sensing, Zhejiang Lab, Hangzhou, Zhejiang, China; ^4^ Cross-Strait Tsinghua Research Institute, Xiamen, China

**Keywords:** photoacoustic imaging, lung imaging, BCI, Monte Carlo simulation, de-artifact, ADF, NLM, phantom verification

## Abstract

Pandemics like COVID-19 have highlighted the potential of Photoacoustic imaging (PAI) for Brain-Computer Interface (BCI) communication and lung diagnostics. However, PAI struggles with the clear imaging of blood vessels in areas like the lungs and brain due to their cavity structures. This paper presents a simulation model to analyze the generation and propagation mechanism within phantom tissues of PAI artifacts, focusing on the evaluation of both Anisotropic diffusion filtering (ADF) and Non-local mean (NLM) filtering, which significantly reduce noise and eliminate artifacts and signify a pivotal point for selecting artifact-removal algorithms under varying conditions of light distribution. Experimental validation demonstrated the efficacy of our technique, elucidating the effect of light source uniformity on artifact-removal performance. The NLM filtering simulation and ADF experimental validation increased the peak signal-to-noise ratio by 11.33% and 18.1%, respectively. The proposed technique adds a promising dimension for BCI and is an accurate imaging solution for diagnosing lung diseases.

## 1 Introduction

The lungs, key organs for gas exchange, are susceptible to harmful substances such as toxic gases, inhalable particles, pathogens, and pollutants during respiration (Lucero and Chan, 2021), which may affect their function and metabolism. In severe cases, exposure to these elements can be life-threatening. The emergence of COVID-19 (Xu et al., 2020) has heightened global concern and the need for lung function assessments to identify pathological changes. While nucleic acid testing can confirm a COVID-19 infection, the pathological characteristics of the disease are mainly determined on the basis of lung conditions. The primary cause of COVID-19-related mortality is abnormal alveolar fluid metabolism, which results in fluid accumulation in the alveoli, known as lung edema (Cui et al., 2021). Edema results from an imbalance in lung tissue fluid formation and reabsorption, which leads to fluid buildup in the lung interstation and alveoli and severely impairs ventilation and gas exchange.

Computed tomography (CT) scans are used to diagnose lung edema in COVID-19 patients, revealing a progression from early insidious interstitial lung edema to later severe alveolar edema (Wiersinga et al., 2020). These symptoms are characteristic of lung viral infections. However, traditional imaging techniques, including CT scans, detect changes in the lung tissue structure and not in the minute capillaries. Moreover, the ionizing radiation from CT renders it unsuitable for long-term use and early pneumonia screening. Therefore, detection technologies with higher resolutions and specificities are urgently required to identify changes safely and effectively in small capillaries. Such advancements could enable earlier intervention and treatment for patients. Additionally, the demand for non-contact assistive communication devices post-viral infection is expanding, leading to a surge in research on multimodal brain-computer interfaces for assistive communication and even language decoding. This includes functional Near-Infrared Spectroscopy devices, which focus on spatial dimensions by detecting changes in blood oxygen levels in the brain to assess neural activity and are suitable for contactless scenarios. However, their resolution and depth penetration for vascular details are relatively low, limiting their efficacy in certain clinical applications.

Photoacoustic imaging (PAI) excels as an innovative imaging modality, facilitating effective imaging of biological tissues up to several centimeters deep. This capability is largely attributed to sound scattering being 1,000 times (Wang and Hu, 2012; Lengenfelder et al., 2019) lower than light scattering. PAI merges the benefits of optical and ultrasound imaging and delivers high resolution, specificity, and remarkable penetration depth (Pang et al., 2022). It has a wide range of applications in both research and clinical stages, enabling anatomical, functional, and metabolic imaging (Yang et al., 2021a). Presently, PAI is being explored for various applications, including the detection of skin melanoma (Breathnach et al., 2018), breast tumors (Xi et al., 2012), and carotid artery blood vessels (Li et al., 2017) and brain functional imaging (Zhang et al., 2018), and has been instrumental in whole-body imaging and disease detection in small animal models (Jeon et al., 2016). Molecules with strong absorption in biological tissues, such as hemoglobin (Guggenheim et al., 2015), melanin (Longo et al., 2017), lipids (Guggenheim et al., 2015), nucleic acids (Yao et al., 2012), and proteins (Zhang et al., 2011) serve as endogenous detection targets for PAI (Zheng et al., 2022). Hemoglobin detection plays an important role in PAI. Recent studies on microvascular detection in small animals or even human brain (Yao and Wang, 2014) have demonstrated the potential of PAI in BCI (Bodea and Westmeyer, 2021) or in detecting capillary changes during early-stage pneumonia, supporting its application in brain and lung detection. Furthermore, several studies based on deep learning for low-light reconstruction (Paul and Mallidi, 2024), as well as research on light-source types and penetration depth (Yang et al., 2024), have paved the way for addressing the issues of light scattering and absorption in tissues, enabling deep organ image reconstruction for the brain or lungs.

However, brain or lung imaging with PAI presents significant challenges owing to the cavity structures, e.g., the ear canals and esophagus or acoustic signals being attenuated by air in the alveoli (Lucero and Chan, 2021). Especially for the lungs, the current results of whole-body imaging in small animals suggest that optical path imaging can only discern the outer contour of the lungs, failing to reveal specific internal details (Raes et al., 2016). This limitation arises from the unique structure of the lungs, which includes flexible lobe tissues, blood vessels, the trachea, and air. Variations in sound velocity passing through pulmonary tissue and air, which cause reflection and attenuation, significantly affect acoustic signal transmission. These factors substantially hamper the transmission of photoacoustic signals (Gröhl et al., 2021). Therefore, investigating the generation and transmission of photoacoustic signals in tissues with cavity structures is crucial for advancing the application of PAI in lung imaging.

In addition to this pioneering work on cavity-structure artifact generation and transmission using simulation and experimental validation, our study includes the selection of appropriate filters for artifact removal in PAI. We focused on artifacts from air tubes, line artifacts arising from sound wave superposition, and background noise due to uneven light source distribution. Although Gaussian filters are widely used, including for functional MRI (Ashburner and Friston, 2000), their main drawback lies in edge blurring caused by the averaging of pixels over dissimilar patterns. To circumvent this, edge-preserving filters such as the anisotropic diffusion filter algorithm (hereafter ADF) have emerged as a preferred alternative (Perona and Malik, 1990; Gerig et al., 1992; Samsonov and Johnson, 2004). ADF effectively reduces simple model cavity structures imaging artifacts caused by ultrasound wave fluctuations yet is ineffective for noise attributed to uneven light distribution. Conversely, the non-local means filter algorithm (hereafter NLM) employs a noise reduction strategy based on the similarity between image regions (Manjón et al., 2008), making it particularly effective for enhancing continuous vascular structures in PAI and reducing noise. This capability makes NLM highly suitable for correcting artifacts in complex PAI scenarios.

However, the application in PAI of neither the ADF nor NLM filters has been extensively studied (Coupé et al., 2009; Guezzi et al., 2022), presenting a significant opportunity for future research to investigate more effective artifact correction strategies for PAI. Furthermore, the work of Steven Guan et al., which successfully implemented a Fully Dense U-Net for artifact removal in sparse 2-D photoacoustic tomography, underscores the potential of deep learning in this field (Guan et al., 2019). While there are concerns about interpretability and the scarcity of data samples, research continues to explore potential clinical applications.

Therefore, we investigated the generation and transmission of photoacoustic signals in cavity-structure tissue, as well as the effectiveness of high-interpretability, low-data-demand ADF and NLM artifact-removal algorithms. The remainder of the paper is organized as follows: Section 2 discusses materials and methods; Section 3 presents the results of the simulation and physical verification, including the de-artifact algorithms and evaluation parameters. Section 4 discusses the results described in Sections 3, 5 comprehensively reviews the overall experiment.

## 2 Materials and methods

Herein, we present a detailed exploration of various aspects related to cavity structures PAI simulation, encompassing physical verification, detailed descriptions of filters, and evaluation metrics.

**FIGURE 1:**
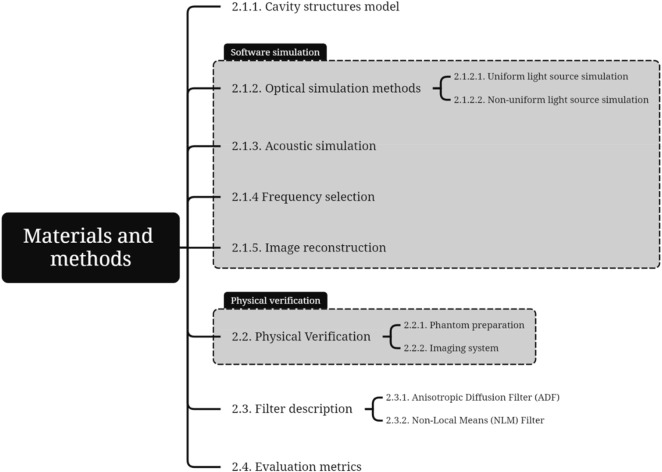
Materials and methods mind map.

Section 2.1 is dedicated to the PAI simulation of cavity structures. It includes the creation of a cavity structures model (Section 2.1.1), outlines optical simulation methods (Section 2.1.2), discusses acoustic simulation (Section 2.1.3), and details image reconstruction techniques (Section 2.1.5). Section 2.2 focuses on the methods used for physical verification. The description of filters is provided in Section 2.3, with ADF explained in Section 2.3.1 while Section 2.3.2 details the NLM filter. Section 2.4 discusses the evaluation metrics used to assess imaging quality. To facilitate understanding, Figure 1 mind maps this section.

### 2.1 Cavity structures PAI simulation

#### 2.1.1 Cavity structures model

**FIGURE 2:**
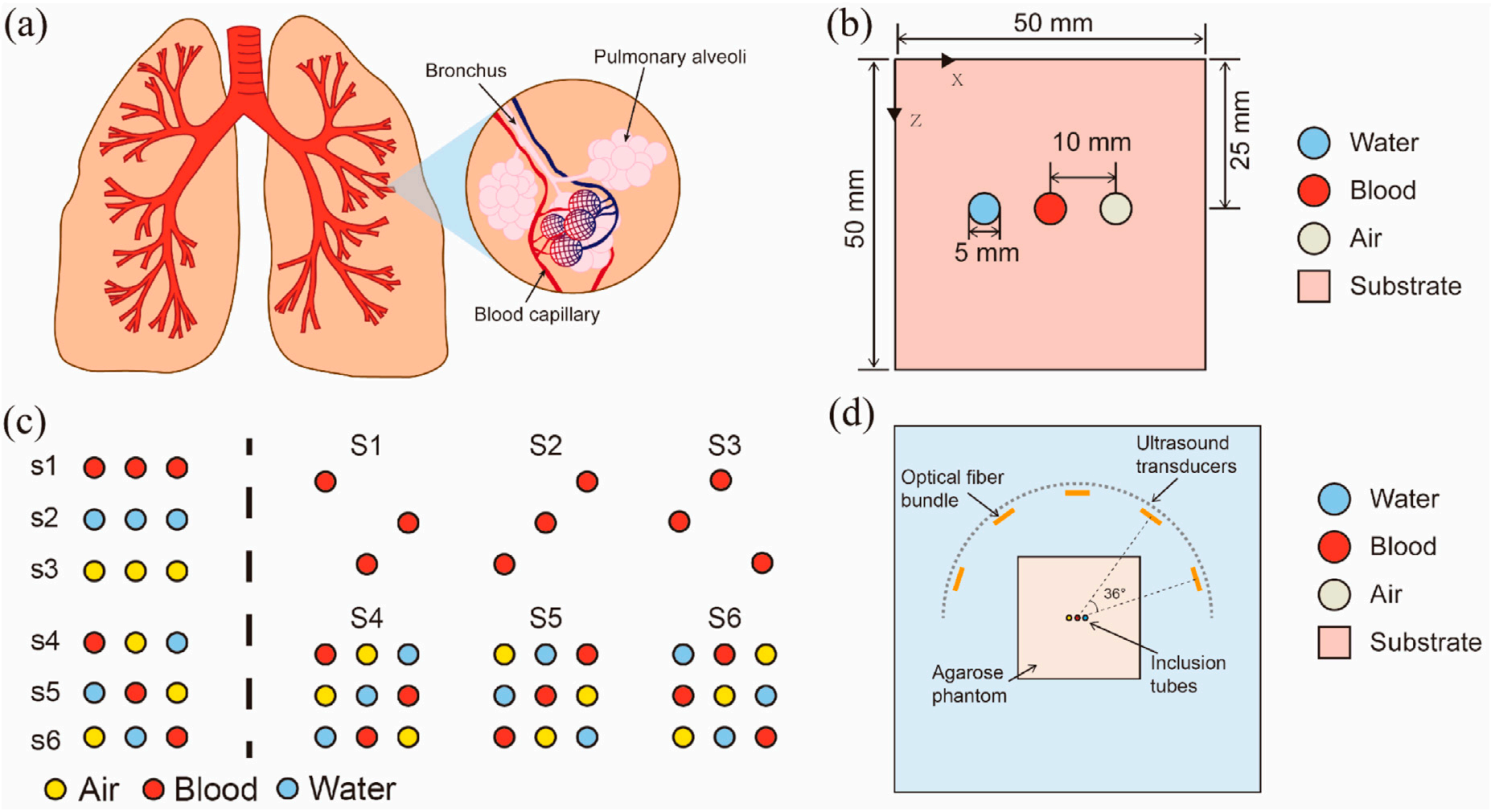
Introduction to a simplified cavity structures model: **(A)** Schematic of the structure of human lungs; **(B)** Simplified cavity structures model used in this experiment; **(C)** Schematic of inclusion distribution in models; **(D)** Schematic of a physical verification system.

Figure 2 shows the simplified cavity structures model used in the experiment. It comprises four parts. All numerical experiments were conducted using MATLAB R2022a on the same computer with an Intel i7-13700K CPU and 64 GB RAM.

Figure 2A shows the lung structure as representative of the organs with cavity structures, emphasizing details of the lung alveoli; the main components, lung lobes, blood, and air, are highlighted.

The cavity structures are simplified in Figure 2B into two key components: a cuboid substrate with circular inclusions, representing muscle, blood, and air. Notably, this model effectively images individual blood tubes, whereas the air and water tubes are negligibly imaged. To investigate the cavity structures’ artifact generation mechanisms and control for the air tubes, a water tube with parameters identical to those of air tubes was included in the cavity-structures phantom design. In this representation, the square symbolizes the substrate, and the circles symbolize water, blood, and air. This simplified model was designed to study and analyze the fundamental mechanisms of sound wave propagation and photoacoustic signal generation in specific regions of the cavity structures. Reducing the complex lung structure to three tubes effectively minimized the computational complexity while controlling the model parameters, thereby concentrating on the key factors in photoacoustic imaging. Although the brain and lung structures are complex, at the preliminary research stage, using three representative tubes served as an initiation point to capture the basic phenomena in cavity structures using PAI.

Figure 2C shows the distribution of inclusions in all models. The left-hand side of the dashed line represents a simple object arrangement, where s1 to s3 illustrate the states of single media (air, blood, water) existing independently, serving as control groups to evaluate the effects of other combinations. Each arrangement from s4 to s6 includes a combination of three different media, systematically positioned to study their interactions and the effects on artifact generation in PAI. The right side of the dashed line employs both control groups (S1-S3) and a 3 × 3 Latin square (S4-S6) to simulate the generation and propagation of artifacts in PAI. This ensures that each experimental condition is evenly distributed (each substance appears in every row and column only once), thereby eliminating the interference of uneven arrangement. The specific arrangements start with blood, air, and water randomly placed in the first row, with the second and third rows generated through cyclic shifting. This arrangement ensures that each medium appears exactly once in every row and column, facilitating a balanced analysis of how different media affect imaging. The representative simple object arrangements of S4-S6 and the matrix object arrangements of S5 are presented in the “*Results*” section.

Last, Figure 2D describes the projection of light sources onto the imaging plane, depicting the general relationship between the light source projection and tube positioning on the plane. A more comprehensive explanation of light source distribution is provided in Section 2.1.2.2.

#### 2.1.2 Optical simulation methods

Herein, we particularly focus on optical simulation methods, distinguishing between simulations with uniform and non-uniform light sources. The values in Table 1 are used for simulations assuming a uniform light source to directly generate initial sound pressure, while the values in Table 2 are used for optical-acoustic simulations with non-uniform light sources and are cross-validated with physical experiments. This distinction is crucial because it influences the accuracy and realism of simulated PAI. The simulations employ a uniform light source, which is associated with consistent light distribution. This approach enables us to study the mechanisms underlying artifact generation in cavity structures PAI more precisely and provides a baseline for assessing the appropriateness of the study’s selected objective evaluation metrics. Additionally, it aids in choosing suitable parameters for the filtering algorithms, thereby enhancing the PAI’s quality and reliability. After designing the evaluation metrics and filtering algorithm parameters, we noticed discrepancies between the results of the uniform light source simulations and physical experiments. To elucidate the reasons for these discrepancies, Section 2.1.2.2 discusses the complexities of non-uniform light source simulations and considers more realistic scenarios with variable light distributions.

**TABLE 1:** Measured and used parameters in uniform light source simulation.

	Agarose	Air	Blood	Water
Light absorption measured at 800 nm	0.056362	0.015132	2.994754	0.002734
Parameters used during the simulation μa (1/cm)	0	0	3	0

Note: All parameters are rounded to one decimal place.

**TABLE 2:** Optical properties of cavity structures tissues at an optical wavelength of 800 nm.

Tissue layers	Absorption coefficient μa (1/cm)	Scattering coefficient, μs (1/cm)	Anisotropy factor, g	Refractive index, n
Background	0.0001	0.1	1.0000	1
Water	0.06	1	0.99	1.3
Blood	2.38	522	0.9	1.4
Air	0.001	347	0.001	1.0

Note: The optical parameters of blood were arterial blood at 800 nm (Li et al., 2011). The optical parameters of water (Liu et al., 2018). Air’s absorption is defined as 0.001 cm^−1^. based on high transmittance (Wang et al., 2021). Atmospheric molecules are considered to be symmetrically scattered; therefore, the scattering anisotropy is close to zero. The atmospheric refractive index was 1.0 with one decimal place reserved (Penndorf, 1957). The scattering coefficient of clean air is measured at 750 nm (Wu et al., 2004).

Selecting a wavelength of 800 nm for light absorption and a Gaussian beam in PAI are strategic choices with several advantages. Being situated in the near-infrared spectrum, this wavelength penetrates deeper into tissue and reduces scattering. It effectively targets vital chromophores such as oxyhemoglobin and deoxyhemoglobin, which is essential for vascular imaging and oxygenation assessment. Importantly, at 800 nm, light is minimally absorbed by air or water, which reduces interference from non-target elements. This feature, along with the safety of NIR light and its compatibility with current imaging systems, makes 800 nm the ideal choice for accurate and efficient PAI. However, to generate the Gaussian beam, the laser of the physical object must pass through a fiber bundle. Its diffusivity is similar to that of a common Gaussian light source. Moreover, the simplicity of the Gaussian beam parameters (waist radius) reduces the difficulty of explaining the relationship between the uniformity of the light source and the imaging effect (Yang et al., 2021b; Yang et al., 2024).

This section intends to elucidate how these two simulation approaches affect the PAI of cavity structures and underscores the importance of considering both in comprehensive research.

##### 2.1.2.1 Uniform light source simulation

To enhance the PAI’s accuracy and minimize artifacts from uneven light distribution, we adopted a method based on the linear relationship between initial sound pressure and optical absorption, grounded in the fundamentals of the photoacoustic effect. The relationship between the initial sound pressure $P_{0}$ and the optical absorption coefficient $\mu_{a}$ can be expressed as:

$$P_{0}=\Gamma \cdot \eta \cdot \mu_{a}\cdot F,$$ (1)

where Γ is the photoacoustic conversion efficiency, η is the heat transfer efficiency and F is the optical fluence. This implies a linear correlation between initial sound pressure and light absorption under our experimental conditions.

**FIGURE 3:**
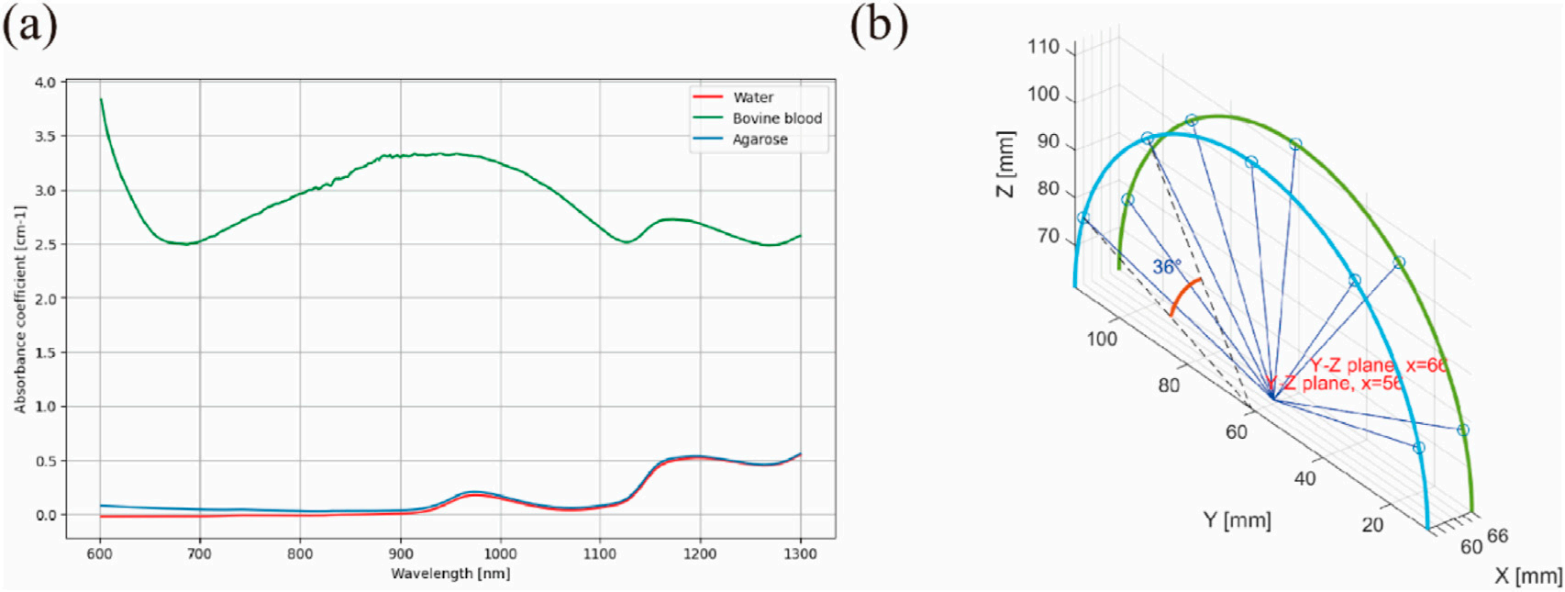
Optical simulation method: **(A)** Variation of the absorption coefficient of main objects; **(B)** Laser emission directions.

The optical absorption coefficients were calculated from absorption spectra measured using a UV-VIS-NIR Spectrophotometer (UV-3600 Plus, SHIMADZU, Japan). Measurements in the sphere absorbance module were conducted using a photometric integrating sphere with an inner diameter of 60 mm, using standard PMT/InGaAs/PbS detectors to ensure a uniform distribution of light in the measurement cavity, thereby reducing the potential impact of non-uniform light absorption on optical fluence F. Each measurement began with auto-zeroing the baseline to 100% transmittance (0 absorbance) and calibrating the background using an empty cuvette. The detection quartz glass cuvette had a transmittance length of 10 mm, with spectra collected from 300 to 1,300 nm at 1 nm intervals. The measured and used parameters are shown in Figure 3A; Table 1.

##### 2.1.2.2 Non-uniform light source simulation

Gaussian light sources were selected as representative for validating that the observed changes in the results of physical verification were not due to the design of the filter parameters and to further investigate the effect of uneven light source distribution on artifact removal in the PAI of cavity structures.

This light source had a waist radius varying from 1 to 20 mm in 1 mm increments, enabling the simulation of the effect of light source distribution on the artifact removal algorithm.

The simulation of optical fluence and absorption was performed in a 3D space using the Monte Carlo method, facilitated by an open-source MATLAB toolbox named “mcxlab” (Yu et al., 2018). A simulated 12 cm × 12 cm × 12 cm geometry was established, within which a 5 cm × 5 cm × 5 cm agarose block containing embedded sealed tubes of 1 mm diameter and 5 cm length was placed to emulate cavity structures tissues. The light source, defined as a Gaussian shape, was illuminated from five directions, each spaced at a uniform angle of 39°. The width of each laser beam was set to 1 mm, with a 10 mm length for the laser source. The total photon number for simulation was set to 10^8^.

The ending time and time-gate width of the simulation were both set to 10^–10^ s, with the laser emission position shown in Figure 3B. The optical properties of the cavity structures tissues at the 800 nm wavelength are shown in Table 2.

The optical fluence was computed using this simulation model. Subsequently, the optical absorption, denoted as *A*, was computed according to Equation 1, identical to that in Section 2.1.2.1.

#### 2.1.3 Acoustic simulation

After the optical simulation to obtain the initial PA signal, we acoustically simulated how a PA wave is propagated and received. This simulation used the k-space pseudo-spectral method, facilitated by the MATLAB k-wave toolbox (Treeby and Cox, 2010).

A 2D half-ring transducer array comprising 128 elements was evenly distributed with a radius of 55 mm to capture the acoustic signals. To account for acoustic heterogeneity, we included the air region (sound speed: 340 m/s; density: 1.2 kg/m^3^) and the other regions (water, blood, and substrate) using the parameters for water (sound speed: 1,500 m/s; density: 1,000 kg/m^3^). The initial pressure for the simulation was calculated on the basis of the optical absorption derived from the optical simulations. A 121 × 121 grid with a 1 mm pitch was used for the calculations, and 4,096 samples were computed at each transducer location, with a temporal resolution of 12.5 ns.

It is important to note that acoustic attenuation was not explicitly modeled in this study. The simulation parameters were chosen to provide a baseline understanding of wave propagation without the inclusion of energy loss due to acoustic attenuation.

#### 2.1.4 Frequency selection

As the PAI simulation of cavity structures differs slightly from that of substantive organs, wherein the sound velocity changes insubstantially, the speed of sound in air considerably affects the size of the simulation frequency, depth, and grid size.

Ultrasonic transmission between 10 kHz to approximately 1 MHz can aid in detecting changes in air and fluid in the thorax owing to their distinct acoustic properties (Rüter et al., 2010). Additionally, ultrasound frequencies between 1 kHz and 10 kHz cannot effectively penetrate the thorax. Frequencies ranging from 10 to 750 kHz can penetrate the human thorax during expiration (Zhang et al., 2024). Because the ultrasonic speed in air is approximately 1/5th of that in water, the maximum frequency supported by ultrasonic waves in air with the same simulation grid size is close to 1/5th of that in water. To retain more high-frequency signals and accurately capture the expected reflection and refraction on high-acoustic impedance interfaces, we selected 750 kHz as the highest supported frequency in water.

Subsequently, the imaging depth could be approximately calculated based on the maximum supported frequency using ${\text{Depth}}_{\max}=\frac{N_{s}∙c}{f_{\max}}$ . Given the speed of sound in air (c = 340 m/s) and the number of sampling points ( $N_{s}=4096$ ), with the maximum supported frequency set at $f_{\max}=750\;KHz$ , wave phenomena can be accurately simulated up to a depth of 1.857 m which is suitable for imaging the brain or lungs.

Having established the maximum imaging depth, we now turn to the critical task of determining the appropriate grid size. Calculating the grid size on the basis of maximum supported frequency involves two approaches: the wavenumber vector and the Nyquist sampling theorem. These ensure the simulation’s accuracy while accommodating the highest frequency of interest within the physical medium. The methodologies are unified under the principle that the spatial step size ( Δx ) must be sufficiently small to resolve the shortest wavelength ( $\lambda_{\min}$ ) corresponding to the maximum frequency ( $f_{\max}$ ) in the medium with sound speed c . The optimal spatial step size is derived by:

$$\Delta x\leq \frac{c}{2f_{\max}}$$ (2)

Wavenumber Vector Approach: This method allows the maximum resolvable wavenumber ( $k_{\max}$ ) to be calculated based on the spatial step size ( Δx ), employing the relationship $k_{\max}=\frac{\pi }{\Delta x}$ . Subsequently, the maximum supported frequency is determined by relating $k_{\max}$ to the sound speed of the medium ( c ), given by $f_{\max}=\frac{k_{\max}∙c}{2\pi }$ , which simplifies to Equation 2 upon substitution. Both methods converge on the identical requirement for Δx , highlighting the fundamental physical and mathematical consistency underlying spatial sampling in wave propagation simulations. Given sound’s speed in air as ( c=340 m/s ) and a maximum frequency of interest ( $f_{\max}=750\;kHz$ ), the spatial step size should not exceed 0.226 mm if the wave phenomena in a 120 mm × 120 mm domain are to be accurately simulated. Therefore, we used a 120 × 120 grid with a 1 mm pitch for the calculations.

#### 2.1.5 Image reconstruction

The delay and sum (DAS) algorithm was selected for image reconstruction. Notably, to ensure that the frequency of the simulation was consistent with that of the physical reconstruction, we performed 750 kHz low-pass filtering on the sampled data before using the DAS algorithm.

For image reconstruction, all parameters of the sensor in the simulation must be consistent with the real shape, size, center frequency, and bandwidth. The speed of sound in water was defined as 1429 m/s , whereas that in air was defined as 430 m/s to accommodate the delay. In addition to the density of the air pipe, the density of water is $1000\;kg/m^{3}$ , and the air pipe density is $1.2\;kg/m^{3}$ . A 1,200 × 1,200 grid with a 0.1 mm pitch was selected for DAS reconstruction.

### 2.2 Physical verification

#### 2.2.1 Phantom preparation

Phantoms were constructed for image acquisition using a PAI system. The materials selected for the phantom included water, bovine blood (HQ60089, EDTA anticoagulant, Hongquan Biological Technology Inc., Guangzhou, China), agarose (CAS: 9012366, Aladdin, Aladdin Biochemical Technology Co. Ltd., Shanghai, China), and polytetrafluoroethylene (PTFE) tubes (Liangqi Inc., Shanghai, China) with an inner diameter of 0.5 mm and an outer diameter of 0.9 mm. Owing to market availability, we opted for a tube diameter of 0.9 mm instead of the ideal 1 mm. The mold for the phantom was a 5 cm × 5 cm × 5 cm cubic box with an opening on top, fabricated using a 3D printer.

The PTFE tubes were cut to a length of 3 cm and filled with either water, air, or blood. The tube ends were sealed with a gel. The agarose solution, prepared from a 1 mg: 100 mL mixture of agarose powder and pure water, was heated in a microwave until boiling and became clear. The solution was then kept warm in an oven at 50°C. Once all components were ready, the agarose solution was poured into the mold in layers to the required height, based on the structure of the phantom.

**FIGURE 4:**
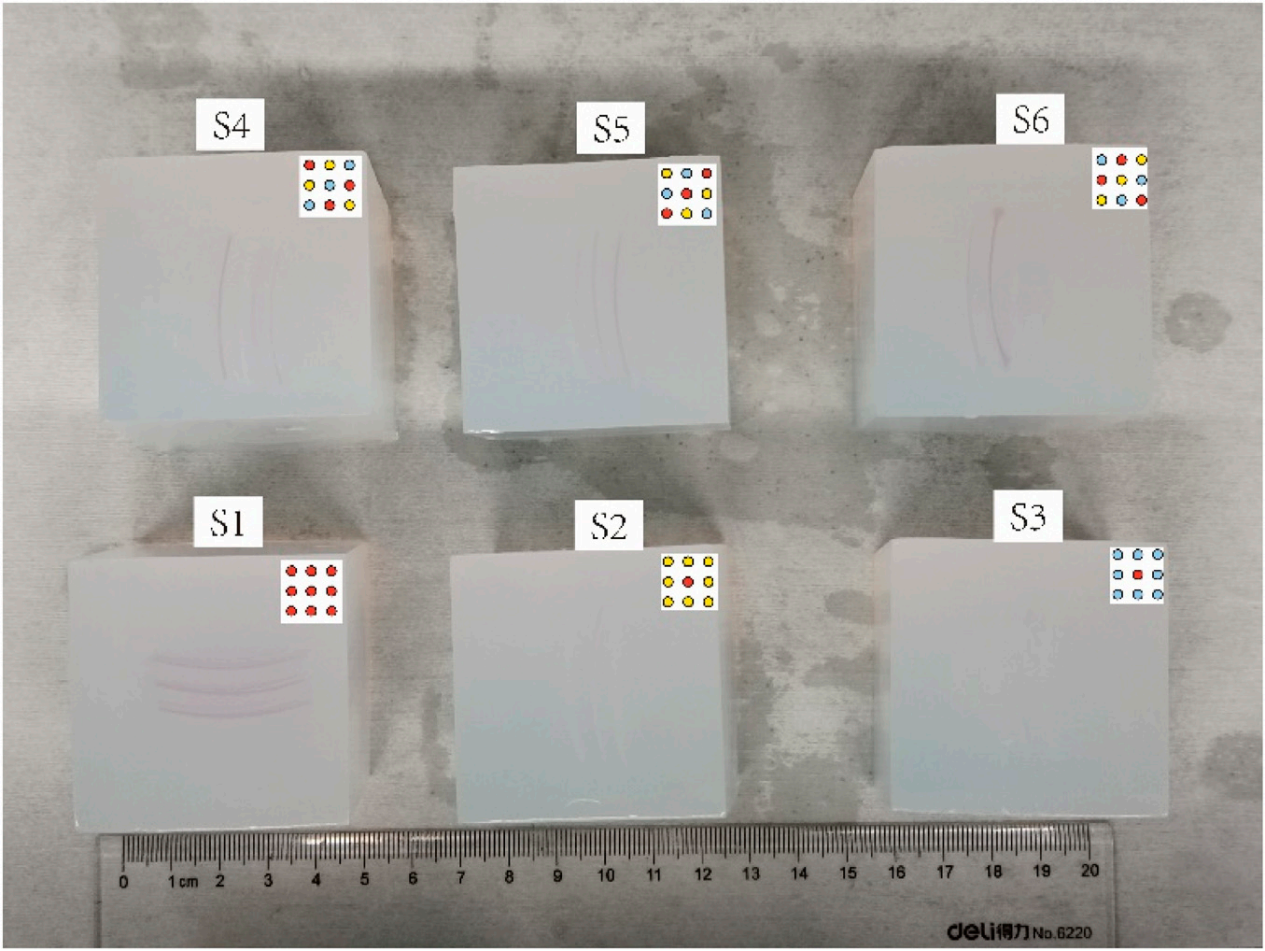
Phantom cube (Top view).

The tubes were placed while pouring and solidifying the agarose solution. After solidification, the phantom was removed from the mold, as shown in Figure 4.

#### 2.2.2 Imaging system

**FIGURE 5:**
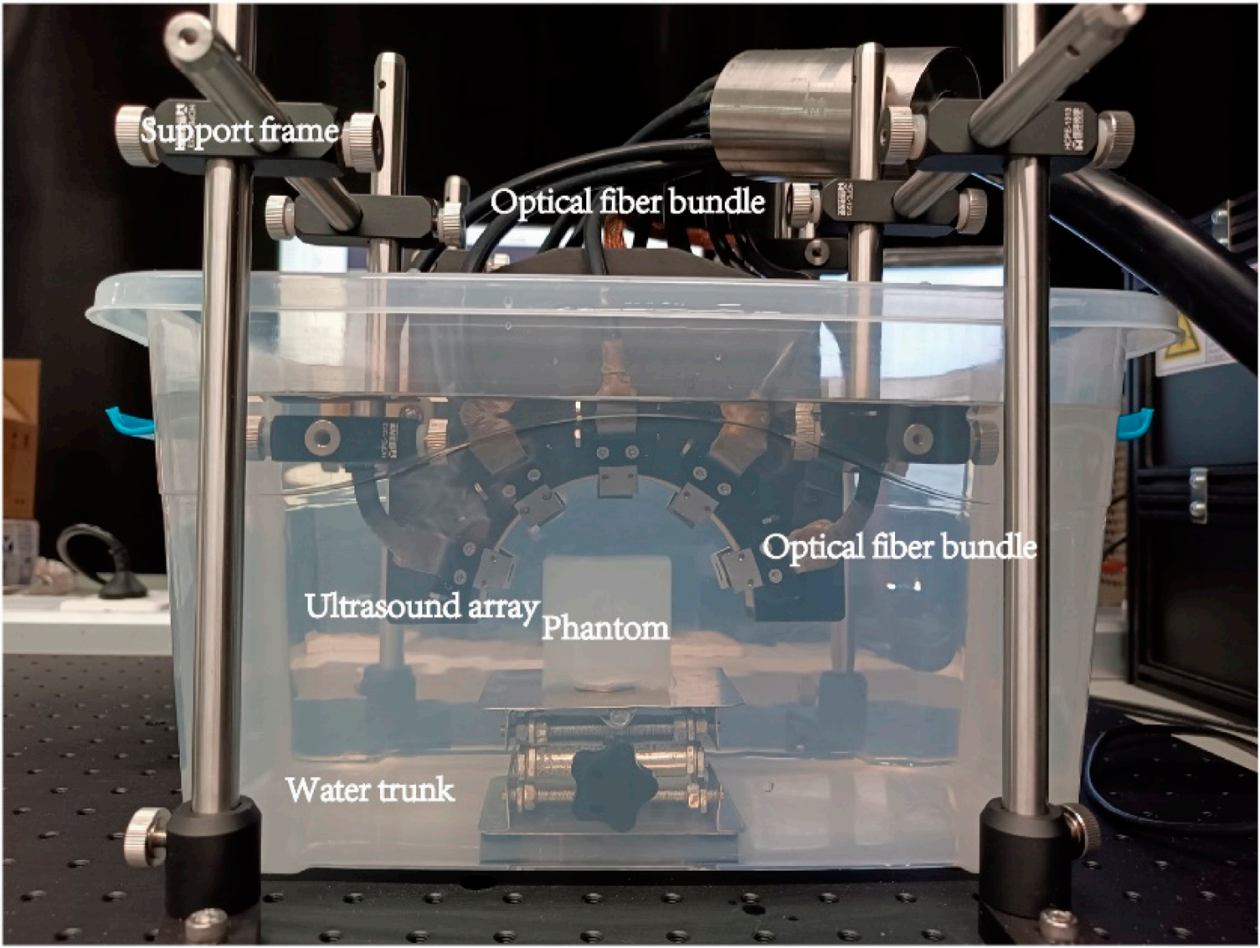
Ultrasonic probe and light source physical arrangement.

**FIGURE 6:**
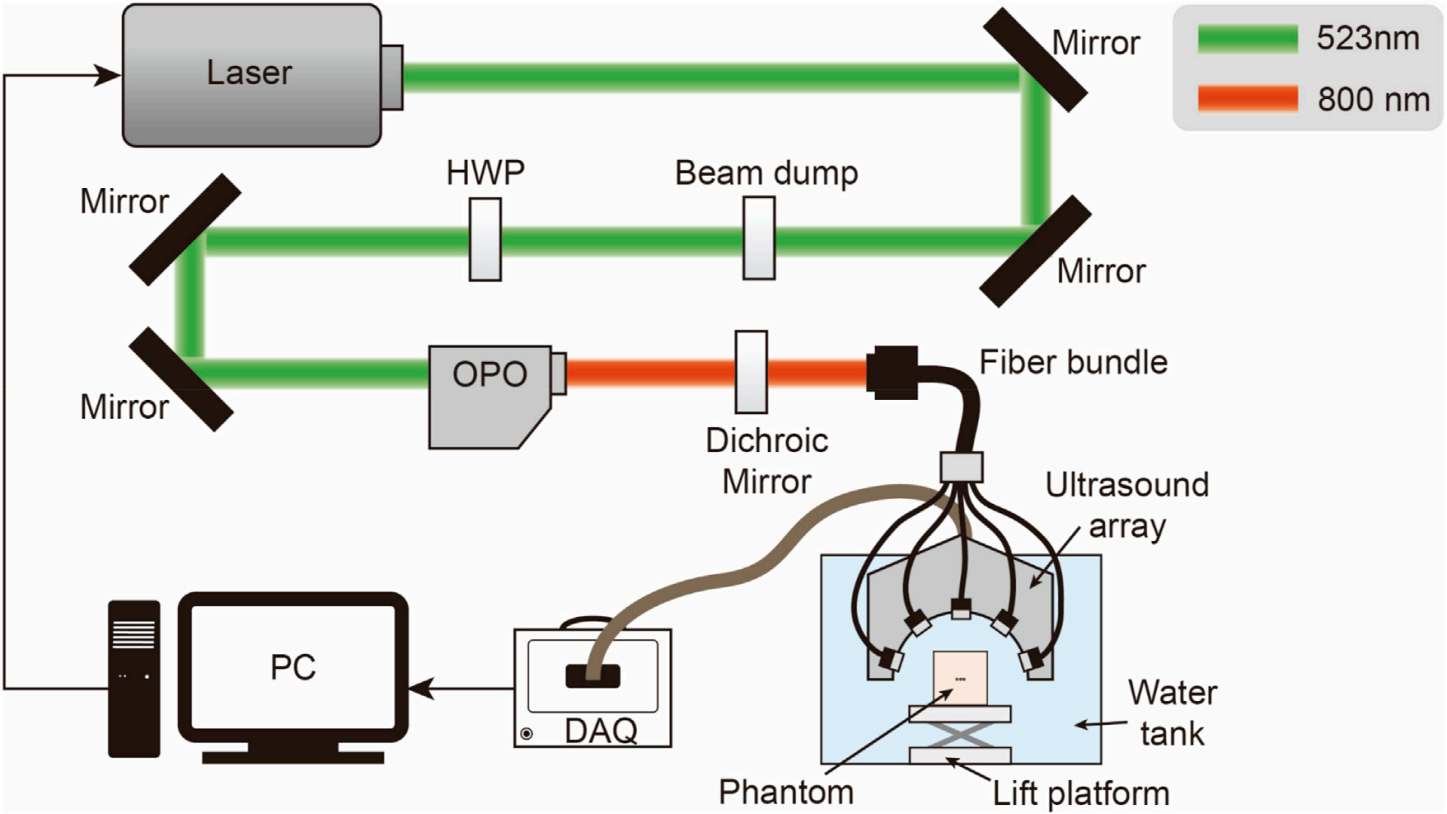
Schematic of the PACT system configuration: HWP, half-wave plate; OPO, optical parametric oscillator; DAQ, data acquisition module, PC, personal computer.

The physical equipment and its structure diagram are shown in Figures 5, 6. The PACT system comprises: an illumination laser, an optical parametric oscillator (OPO), a half-ring ultrasonic transducer array, a data acquisition system, and a computer. For photoacoustic excitation, a 523 nm optical beam from a laser (Nimma 900; Beamtech Inc., Beijing, China; 10 Hz pulse repetition rate; 8 ns pulse width) was modulated to the required wavelength using an OPO (BB-OPO-532; Deyang Tech Inc., Zhejiang, China; output wavelengths ranging from 680 nm to 960 nm) and transmitted through custom optical fiber bundles (Qingpai Tech. Inc., Beijing, China). Initially, a mirror reflected the light beam, altering its propagation direction before entering the OPO. A beam dump was positioned on the optical path to absorb the 532 nm light reflected from the OPO.

A half-wave plate then adjusted the polarization of the 532 nm laser beam to match the polarization state required by the OPO. Subsequently, any remaining 532 nm light was redirected by a dichroic mirror before entering the fiber bundle.

In the PAI system, a custom half-ring ultrasonic transducer array (Qingpai Tech. Inc., Beijing, China; 128 elements; 110 mm ring diameter; 5 MHz central frequency) was used for 2D panoramic detection. A semicircular array was selected because of its unique geometric layout, which enabled a more precise exploration of the principles underlying artifact formation. Particularly when analyzing artifacts caused by internal structures such as ducts, a semicircular array mitigates the issue of linear artifacts potentially being mistaken for the shape of the probe. Additionally, distinguishing imaging artifacts of circular arrays from the anisotropic artifacts of circular tubes can be challenging. Therefore, the selection of a semicircular array was crucial for accurately identifying and interpreting specific features in the imaging results. Furthermore, this transducer array was connected to a data acquisition module (Marsonics128; Langyuan Tech. Inc., Tianjin, China; 128 Channels) capable of 80 MSPS and a maximum amplification of 128 dB.

### 2.3 Filter description

In the PAI of cavity structures, noise can arise from various factors, including the uneven distribution of light and the effect of acoustic impedance on sound wave propagation. This noise is primarily multiplicative, characterized by stronger noise in brighter areas of the image and weaker noise in darker areas. Handling multiplicative noise is more complex than handling additive noise because it varies with the image signal strength. To address such noise, we selected ADF, which concentrates on local image features, and NLM, which is suitable for cavity-structure images with repetitive patterns and structures. Both algorithms are also effective in dealing with additive noise, as they can smooth interior regions while preserving edges (Yu and Acton, 2002).

#### 2.3.1 Anisotropic diffusion filter

This type of algorithm simulates natural diffusion processes, dynamically adjusting the diffusion process based on the local characteristics of the image, to maintain the edges and details of the image while eliminating noise. In the approach introduced by Perona and Malik (Perona and Malik, 1990), an anisotropic coefficient is used to stop diffusion across image edges. This is expressed as:

$$\left\{\begin{array}{l} \frac{\partial I}{\partial t}=\mathrm{div}\left[c\left(\left.\left|\nabla \right.I\right|\right)\nabla I\right]\\ I\left(t=0\right)=I_{0} \end{array}\right.$$ (3)

$$\left\{\begin{array}{l} c\left(x\right)=\frac{1}{1+{\left(x/2\right)}^{2}}\\ c\left(x\right)=\exp\;\left[-{\left(x/k\right)}^{2}\right] \end{array}\right.,$$ (4)

where ∇ is the gradient operator, div represents the divergence operator,   denotes magnitude, $c\left(x\right)$ is the diffusion coefficient, and $I_{0}$ is the initial image. Two diffusion coefficients are suggested in Equation 4: where k is a parameter indicating edge magnitude.

In anisotropic diffusion, the gradient magnitude is used to identify image edges or boundaries as step discontinuities in intensity (Yu and Acton, 2002). If ∇I>k , then $c\left(\left|\nabla I\right|\right)\to 0$ , resulting in an all-pass filter; if ∇I<k , then $c\left(\left|\nabla I\right|\right)\to 1$ , leading to isotropic diffusion (Gaussian filtering) (Bavirisetti and Dhuli, 2015). The discrete form of this Equation 3 is given by Equation 5

$$I_{s}^{t+∆t}=I_{s}^{t}+\frac{∆t}{\left|n_{s}\right|}\sum_{p\in \eta }c\left(\nabla I_{s,p}^{t}\right)\nabla I_{s,p}^{t}$$ (5)

where $I_{s}^{t}$ is the discretely sampled image, s denotes the pixel position in a discrete two-dimensional grid, ∆t is the time step size, $n_{s}$ represents the spatial neighborhood of pixel s , and $\left|n_{s}\right|$ denotes the number of pixels in the window (usually four, except at image boundaries) (Yu and Acton, 2002). The difference between pixel intensities is represented as Equation 6

$$I_{s}^{t+∆t}=I_{p}^{t}-I_{s}^{t},\forall p\in \eta_{s}$$ (6)

To effectively implement ADF in practice, these partial differential equations must be digitized.

#### 2.3.2 Non-local means filter

In contrast to ADF, which is dependent on the immediate neighborhood of a pixel, NLM considers the similarity between distant pixels or regions. Fundamentally, it involves replacing the intensity of each pixel with a weighted average of intensities from all other pixels in the image, where the weights are determined by the similarity between pixel neighborhoods (Bhujle and Vadavadagi, 2019). This approach is particularly suitable for images of vascular structures with repetitive patterns. The NLM filtering process is mathematically represented as follows Equation 7 (Manjón et al., 2008; Buades et al., 2011):

$$I^{\prime }\left(p\right)=\frac{1}{C\left(p\right)}\sum_{q\epsilon I}f\left(p,q\right)∙I\left(q\right)$$ (7)

where $I^{\prime }\left(p\right)$ represents the filtered intensity of pixel p , $I\left(q\right)$ is the intensity of a pixel q , and $f\left(p,q\right)$ is a weight function based on the similarity between the neighborhoods of pixels p and q . $C\left(p\right)$ is a normalization factor defined as Equation 8

$$C\left(p\right)=\sum_{q\epsilon I}f\left(p,q\right)$$ (8)

The weight function $f\left(p,q\right)$ is typically defined as an exponentially decreasing function of the Euclidean distance between the intensity vectors of the neighborhoods of p and q . This often includes a Gaussian filtering component (Manjón et al., 2010) Equation 9:

$$f\left(p,q\right)=\exp\left(-\frac{{\left\|v\left(p\right)-v\left(q\right)\right\|}^{2}}{h^{2}}\right)$$ (9)

where $\upsilon \left(p\right)$ and $\upsilon \left(q\right)$ are vectors representing the neighborhoods of pixels p and q , respectively, whereas h is a filtering parameter that controls the rate of decay of the exponential function.

### 2.4 Evaluation metrics

To evaluate the effectiveness of these artifact-removal algorithms in PAI, we employed a combination of subjective and objective assessment methods. Recognizing that objective evaluations may not fully represent the quality of image improvement, (Hore and Ziou, 2010), we aimed to achieve a more comprehensive and in-depth understanding of the true effectiveness of the algorithms by analyzing results from both approaches. Additionally, we adjusted our objective evaluation metrics to account for variations caused by changes in the light-source distribution.

We selected objective evaluation metrics that were statistically used in more than 20% of cases, as identified in a review (Gröhl et al., 2021), along with additional indicators that enhanced visual details and image quality. These include peak signal-to-noise ratio (PSNR), structural similarity index (SSIM), mean square error (MSE), and normalized absolute error (NAE), as deduced in MATLAB^®^ (Cadik and Slavik, 2004; Hore and Ziou, 2010). In contrast to a previous study (Yalavarthy et al., 2021) that used the NLM filter and focused only on decibel improvement, we aimed to compare lift rates between the anticipated imaging, the original image, and filtered image of the anticipated imaging-original image. In essence, these metrics were calculated using anticipated imaging $G_{a}$ and the original image $G_{o}$ . Given the filtered image $G_{f}$ of the original image, the equation for lift rates is in Equation 10:

$$X_{lift\_ratio}=X\left(G_{a},G_{f}\right)-\frac{X\left(G_{a},G_{o}\right)}{X\left(G_{a},G_{o}\right)},$$ (10)

where X represents various evaluation metrics.

These metrics cover various aspects, from pixel-level errors (such as MSE and NAE) to structural and visual quality (such as PSNR and SSIM). This diverse range of metrics ensures a comprehensive evaluation, aiding comparison and highlighting the strengths and potential limitations of ADF and NLM. This comprehensive approach ensures their effectiveness and reliability in practical applications.

An optimal filtering algorithm should yield higher values in PSNR, SSIM while maintaining lower values in MSE and NAE. High PSNR, SSIM scores indicate superior image quality, fidelity, and structural integrity, whereas lower MSE and NAE values signify minimal reconstruction errors and discrepancies compared with those in the original image.

## 3 Results and discussions

This study thoroughly analyzed the generation, propagation, and reconstruction of PA signals, focusing on signals from three objects: blood, water, and gas. The simulation and explanation of artifacts generated by these materials form the core of our analysis, and the results are shown in the following sections.

**FIGURE 7:**
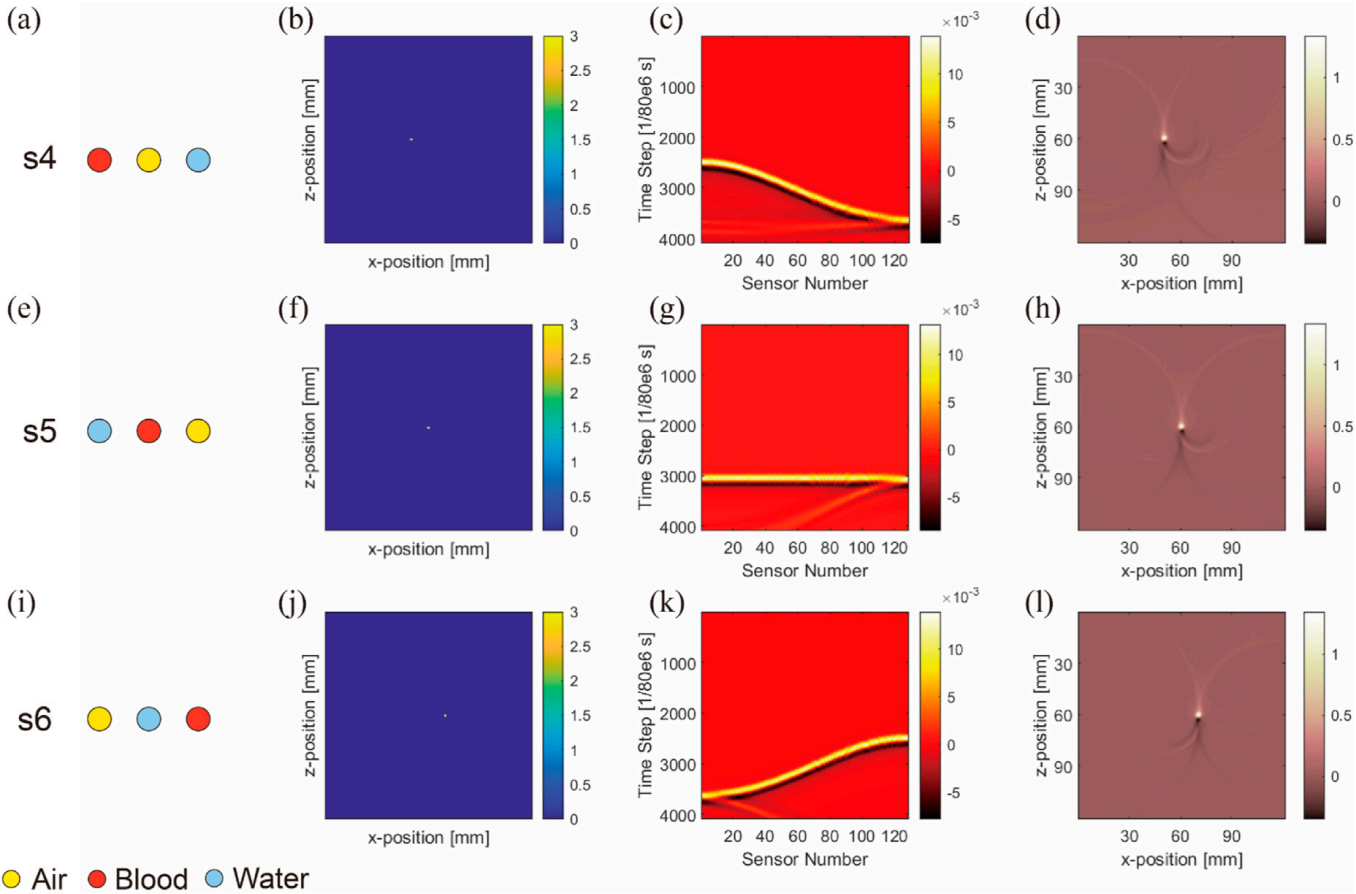
Image reconstruction results of three embedded nonidentical inclusions. Results from the first to fourth columns belong to ideal arrangement **(A,E,I)**, initial sound pressure **(B,F,J)**, sensor data **(C,G,K)**, and image reconstruction result **(D,H,L)**, respectively. Results from the first to third rows belong to blood–air–water **(A–D)**, air–blood–water **(E–H)**, and air–water–blood **(I–L)**, respectively.

It is noteworthy that in this section, as exemplified by Figure 7, the second column (Figures 7B, F, J) displays the distribution of initial sound pressure. These images show the initial sound pressure derived from a linear equivalence based on absorption intensity, thus the represented data are dimensionless. The sensor data shown in the third column (Figures 7 C, G, K), derived from these initial sound pressure data, are also dimensionless. Finally, all result data have been normalized to ensure that they represent relative changes rather than specific physical units, maintaining the consistency of the dataset being dimensionless. This approach not only simplifies the comparison and analysis of data but also helps to more clearly demonstrate the relative differences in how various materials affect photoacoustic signals.

### 3.1 Simulation results

#### 3.1.1 Uniform light source

##### 3.1.1.1 Simple object arrangement (Three nonidentical inclusions arranged horizontally)

Figure 7 shows the initial sound pressure, sensor data, and image reconstruction for three different inclusions within the substrate. The first to third rows correspond to s4-blood–air–water (Figures 7A–D), s5-air–blood–water (Figures 7E–H), and s6-air–water–blood (Figures 7I–L) configurations, respectively.

One significant observation from the simulation is the close relationship between artifact formation and the anisotropy of air inclusions. This becomes particularly evident when comparing air inclusions with water inclusions. Despite their similarity in parameters such as light absorption, they differ in anisotropy. Further analysis revealed that density and the sound speed in the air medium affect acoustic signal propagation. However, a more fundamental factor appears to be the effect of acoustic impedance on sound wave propagation. At interfaces between media with mismatched acoustic impedances, strong sound wave reflections occur, leading to artifact formation.

**FIGURE 8:**
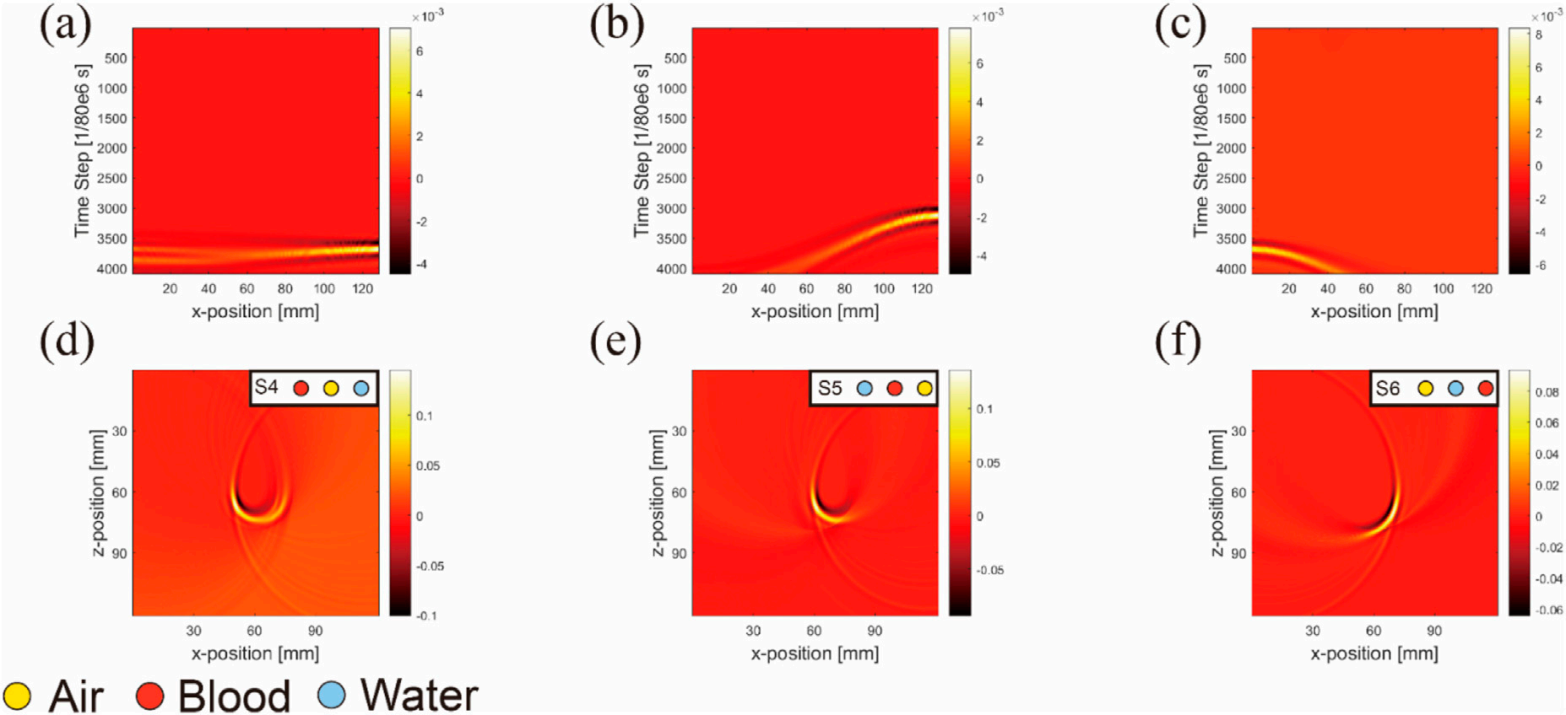
Sensor data **(A–C)** and image reconstruction results **(D–F)** of pure artifact images for three nonidentical inclusions arranged horizontally. (Uniform light source): The columns from left to right are pure artifacts of blood–air–water, air–blood–water, and air–water–blood respectively; the simulation layout conditions are marked at the top right-hand corners of images.

To demonstrate artifact occurrence more clearly, we conducted two types of simulations and subtracted their results, as shown in Figure 8. One simulation included artifacts (with air, water, and blood inclusions) and the other was artifact-free (featuring only blood inclusion). By subtracting these results, we isolated pure artifact images from the sensor data (Figures 8A–C) and image reconstruction results (Figures 8D–F). We observed that in simulations with horizontal inclusion arrangements, the main form of artifacts in anisotropic simulations with air inclusion appeared as a semicircle.

This semicircle was centered at the air inclusion, with its radius being the distance from the blood inclusion to air inclusion, and the blood inclusion being the starting point of the semicircle.

##### 3.1.1.2 Advanced object arrangement (Nine nonidentical inclusions arranged in a matrix)

**FIGURE 9:**
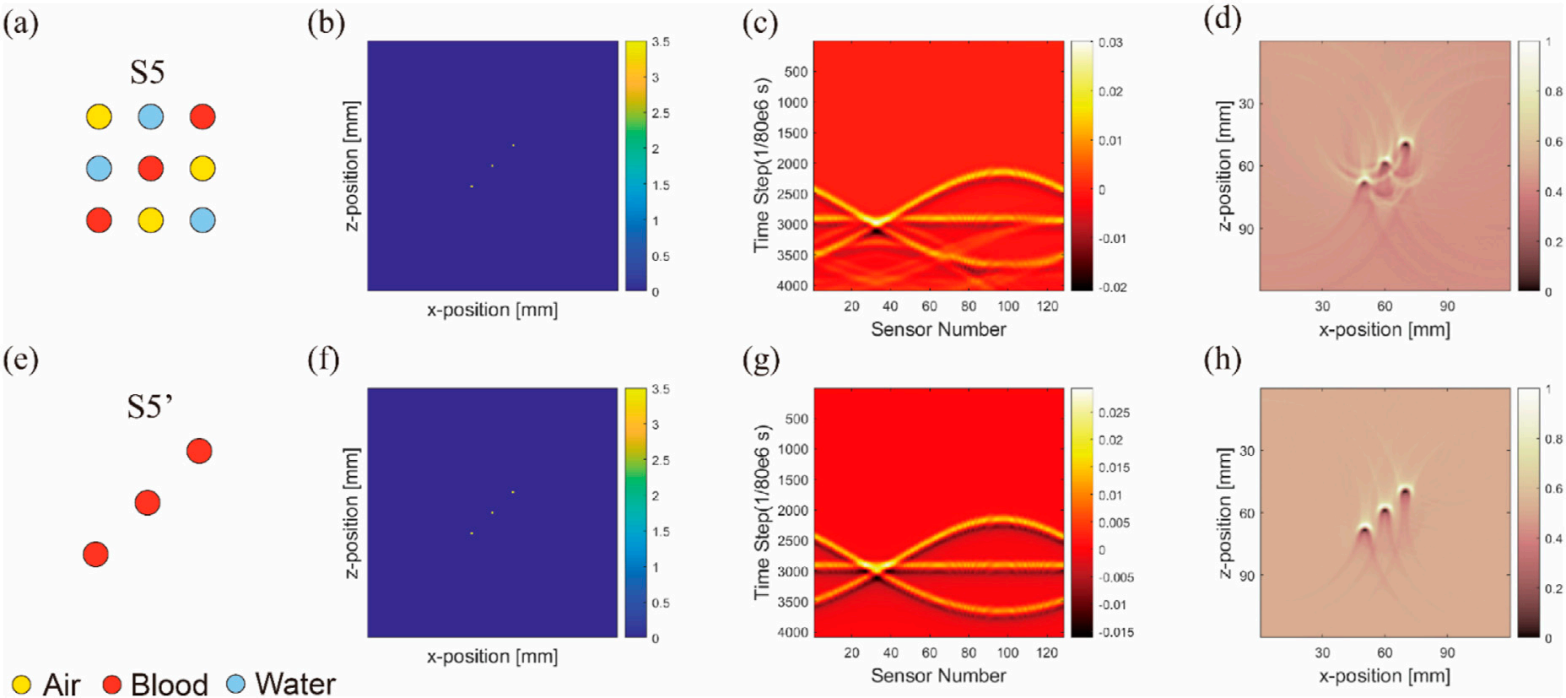
Image reconstruction results of multi-inclusion matrix arrangement model. Results from the first to fourth columns correspond to ideal arrangement **(A,E)**, initial sound pressure **(B,F)**, sensor data **(C,G)**, and image reconstruction result **(D,H)**, respectively. (Uniform light source): results from the first to second rows correspond to three inclusions in matrix arrangement **(A–D)**, and only blood in matrix arrangement **(E–H)**, respectively.

In our model, the arrangement of inclusions was varied to analyze their effect on sensor data and artifact formation. The first row (Figures 9A–D) followed the arrangements of S5, whereas the second row (Figures 9E–H) was set to S5.

**FIGURE 10:**
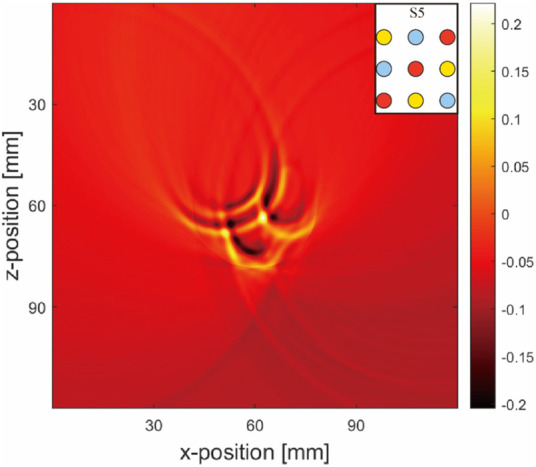
Pure artifact for a multi-inclusion matrix arrangement model (Figures 9D–H): Simulation layout conditions are marked at the top right-hand corners of the images.

A noticeable difference was observed between the sensor data values from the ultrasound transducer elements in simulations that included air tubes (Figures 9A–D) and those without air tubes (Figures 9E–H). By subtracting the results from two DAS algorithms, we isolated pure artifact images (Figure 10). In these images, artifacts from the simulations with air tubes had distinct shapes, forming a semicircle with the air tube at the center. The radius of this semicircle was the distance from the blood tube to the air tube, with the blood tube being the starting point. The direction of this semicircle was determined by the orientation of the sensor toward the air tube.

#### 3.1.2 Physical results

##### 3.1.2.1 Simple object arrangement (Three nonidentical inclusions arranged horizontally)

**FIGURE 11:**
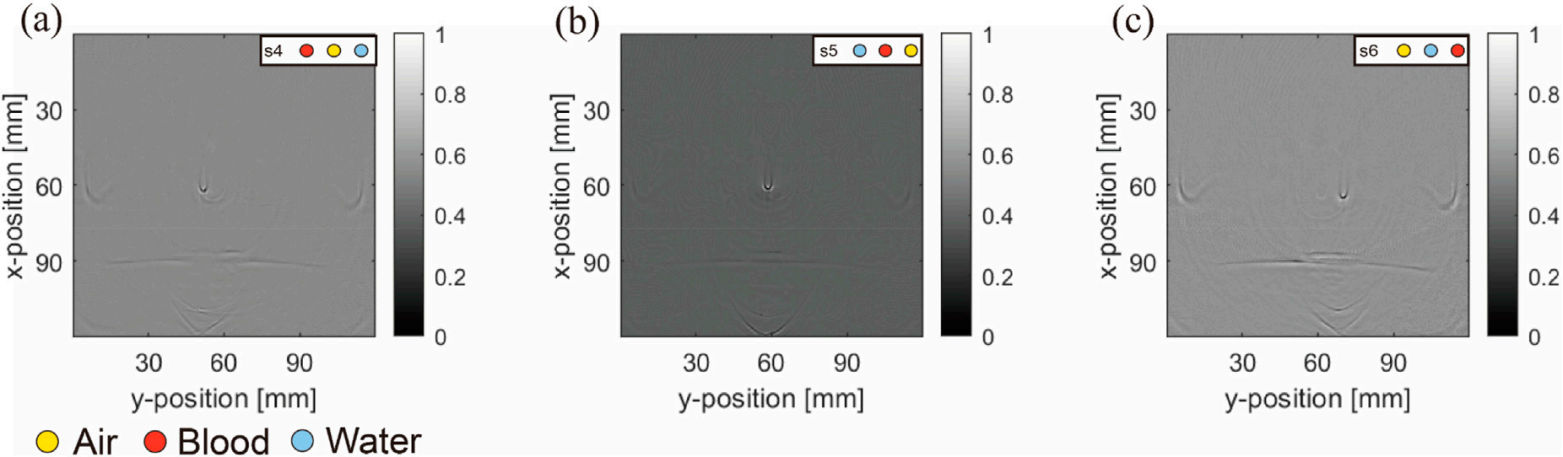
Physical results: Results from **(A–C)** correspond to blood–air–water, air–blood–water, and air–water–blood, respectively. Simulation layout conditions are marked at the top right-hand corners of the images.

Figure 11 shows images reconstructed from the experiments, which show trends similar to the simulated results. The blood tubes exhibit the strongest signal in the images, whereas the signals from air and water tubes are less visible. Unlike the simulated predictions, the images displayed faint signals from the PTFE tubes containing blood, air, water, and artifacts from the holder and fixture in contrast to the background. Furthermore, owing to the limited field of view of the half-ring array, the regions below it had missing information and half-circle artifacts.

In summary, comparing the simulation and experimental results of the model with three object tubes indicated that the blood tube yielded a stronger signal than the air and water tubes. The contrast in the water tubes was the lowest among all groups. Artifacts were notably generated in the presence of both blood and air tubes.

##### 3.1.2.2 Advanced object arrangement (Nine nonidentical inclusions arranged in a matrix)

In the advanced matrix phantom setup, the simulations and experiments revealed more complex reconstructed images, with a higher number of objects leading to increased artifacts. In the pure blood tube model (Figures 9E–H), distinct artifacts around the blood tube were observed, differing from those caused by the acoustic heterogeneity of air. In models with blood tubes surrounded by air or water tubes (Figures 9A–D), the surrounding air tubes significantly deformed the artifacts of the blood tubes, while the deformations arising from the water tubes were relatively minor.

**FIGURE 12:**
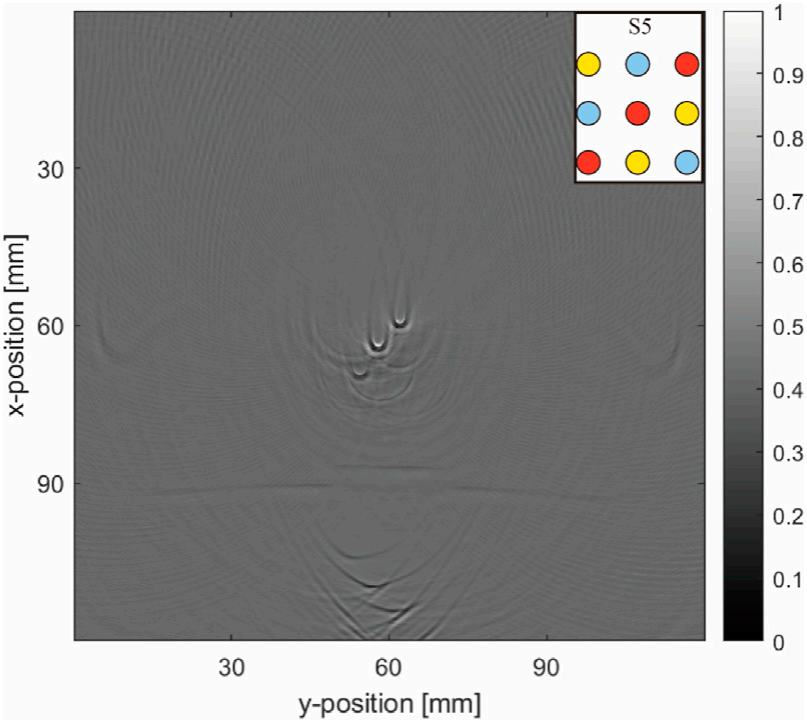
Physical result matrix arrangement. The simulation layout is given at the top right-hand corner of the image.

Artifacts in the blood tubes (Figure 12) appeared as large arc-like intersecting lines. Initially, these artifacts were less noticeable in simple object arrangements owing to the substantial signal intensity of the blood tubes, making them less conspicuous compared with the artifacts caused by air. Consequently, they were ignored in our initial observations. However, in the matrix models, these artifacts became more pronounced and were notably altered by the presence of air tubes.

### 3.2 Artifact removal

The objective evaluation metrics given in Section 3.2 indicate that applying the two algorithms improved the processing performance. Notably, the lower MSE and NAE values correlate with better image quality, suggesting that reductions in these values signify an enhancement in image quality.

#### 3.2.1 Uniform light source artifact removal

The performance of NLM was superior to that of ADF in simulations with simple uniform light source anisotropy arrangements. This is summarized in Table 3.

**TABLE 3:** Objective evaluation results in comparing two artifact-removal algorithms on a uniform light source simulation with a simple and matrix object arrangement base.

	Arrangement	Algorithm	PSNR (%)	SSIM (%)	MSE (%)	NAE (%)
Uniform light source anisotropy simulation	Simple object arrangement	**NLM correction lift ratio**	**11.33**	**1.05**	**−78.38**	**−53.63**
		ADF correction lift ratio	3.60	0.51	−38.56	−21.66
	Matrix object arrangement	**NLM correction lift ratio**	**4.79**	**2.11**	**−44.33**	**−25.40**
		ADF correction lift ratio	4.58	2.04	−42.90	−24.48

Note: Bold black indicates that, according to the selected evaluation index, the algorithm is superior to the other algorithm under the same conditions.

**FIGURE 13:**
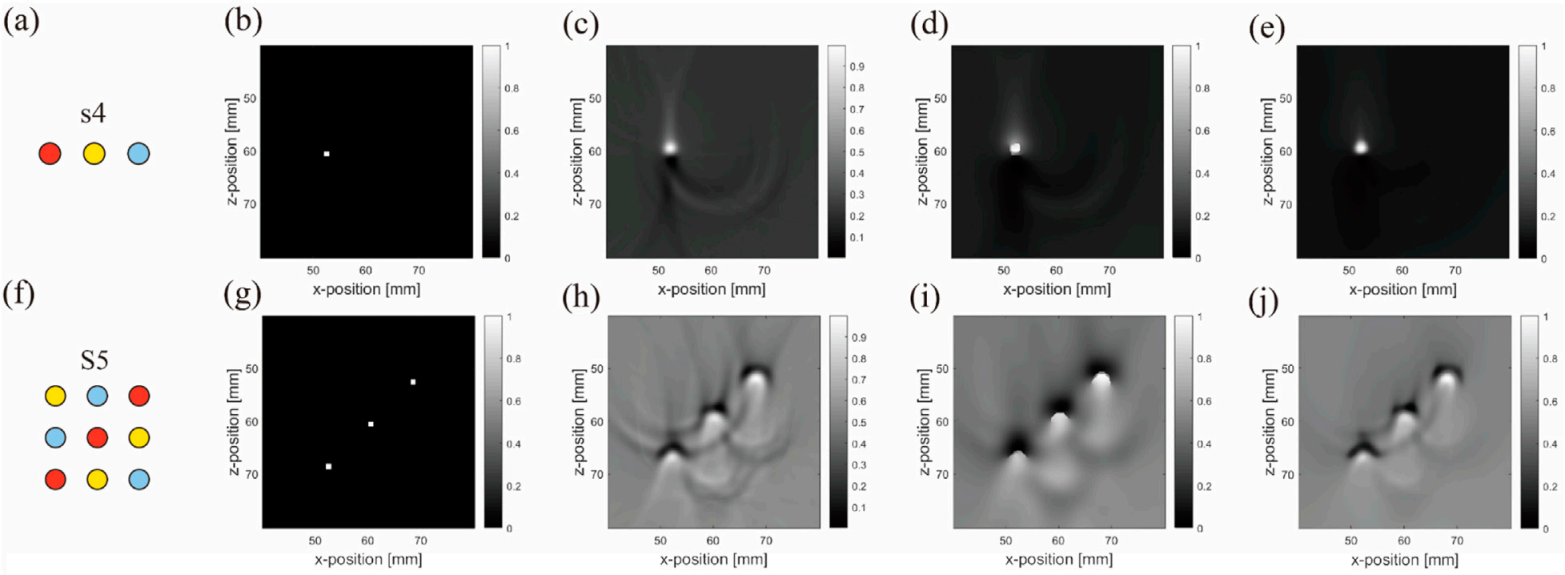
Original and corrected contrast images using two de-artifacting algorithms under uniform light source simulation: **(A–E)** are results from the first to second rows belonging to the blood–air–water (S4) arrangement, and **(F–J)** are three different inclusions in the matrix (S5) arrangement. **(A,F)** are ideal arrangements, **(B,G)** are ideal reconstruction results, **(C,H)** are reconstruction results, **(D,I)** are ADF correction results, **(E,J)** are NLM correction results. Units: millimeters (1 mm).

The objective results demonstrate the superiority of the NLM method in the simulations. It preserved the edges of the blood tubes more effectively than ADF and filtered out background noise caused by the superposition of acoustic characteristics. In particular, the PSNR results were considerably improved, indicating that NLM effectively retained structural content and differentiated between background and object more effectively than ADF. We believe that this observation is inseparable from the characteristics of NLM. In scenarios with simple arrangements, the conditions often exhibit repetitive patterns, which establish favorable conditions for NLM’s filtering capabilities. Considering these findings alongside the image results, our choice of indicators was evidently rational. The correction images (Figure 13) and corresponding subjective results are summarized below.

The subjective results confirm that with uniform lighting, NLM effectively removed background noise and air artifacts from the horizontal arrangement (Figures 13D, I). Although slightly less effective in matrix arrangements, NLM still cleanly processed the background. Overall, NLM outperformed ADF under uniform background or lighting conditions, emphasizing its utility in specific PAI scenarios.

#### 3.2.2 Real experiment’s artifact-removal

Under real experimental conditions, the evaluation results were contrary to those under uniform lighting conditions. Surprisingly, even in a repetitive environment with a multichannel arrangement, where NLM was expected to perform well, its performance remained inferior to that of ADF. This is summarized in Table 4.

**TABLE 4:** Objective comparative evaluation results for the two de-artifacting algorithms in real experiments with simple and matrix object arrangement bases.

	Arrangement	Algorithm	PSNR (%)	SSIM (%)	MSE (%)	NAE (%)
Real experiment de-artifacts	Simple object arrangement	NLM correction lift ratio	15.12	6.14	−83.21	−59.03
		**ADF correction lift ratio**	**18.10**	**6.53**	**−88.20**	**−65.69**
	Matrix object arrangement	NLM correction lift ratio	0.91	0.28	−11.02	−5.57
		**ADF correction lift ratio**	**3.18**	**0.86**	**−33.55**	**−18.56**

Note: Bold black indicates that, according to the selected evaluation index, the algorithm is superior to the other algorithm under the same conditions.

We experimentally investigated the superiority of the ADF method, as demonstrated by the evaluation data, in actual artifact removal. ADF effectively preserves structure and reduces noise. Note that the effect of nonuniform lighting on images can cause greater variations in certain evaluation metrics. In both the simulation and real experimental validation, switching to the superior algorithm (NLM for simulation and ADF for practical) led to notable improvements in the commonly used PSNR metric (11.33% in the simulation and 18.1% in practice) and as the most significantly changed metric, MSE decreased to 78.38% in the simulation and 88.20% in practice. The experimental validation showed better results than the simulations, owing to the uneven lighting conditions also being eliminated by the filtering algorithms.

**FIGURE 14:**
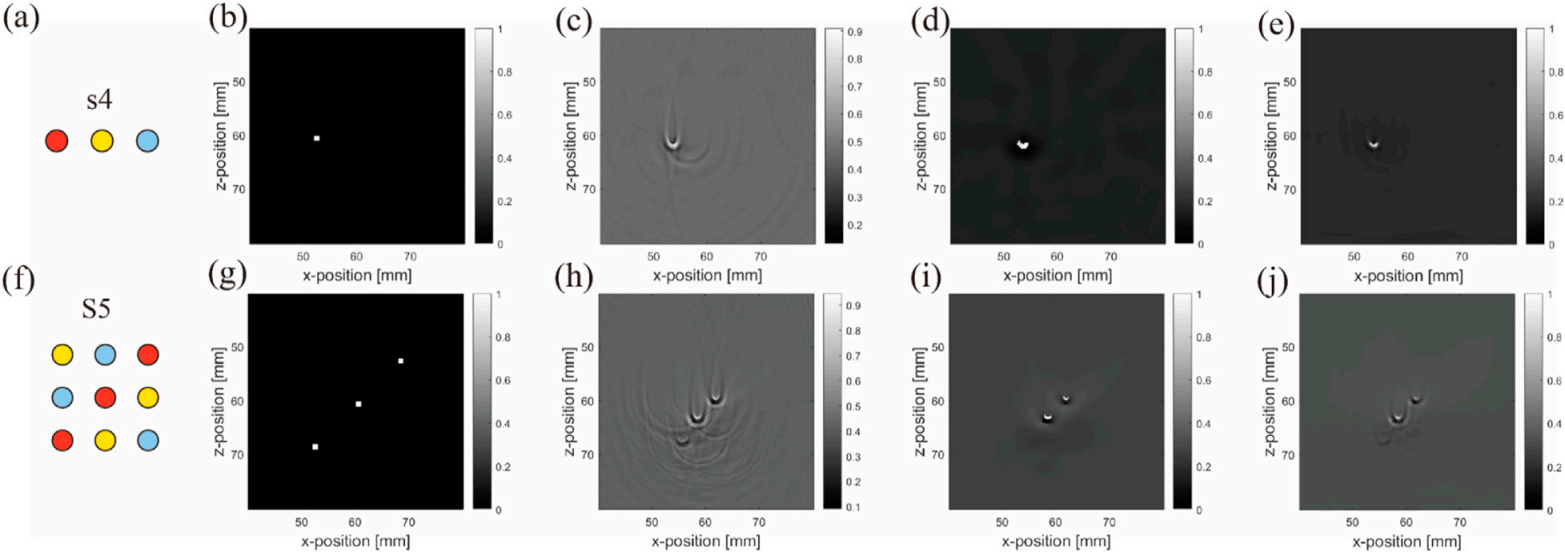
Real experiment: Original and corrected contrast images using two de-artifacting algorithms: **(A–E)** results from the first to second rows belonging to the blood–air–water (S4) arrangement, and **(F–J)** are three different inclusions in the matrix (S5) arrangement. **(A,F)** are ideal arrangements, **(B,G)** are ideal reconstruction results, **(C,H)** are reconstruction results, **(D,I)** are ADF correction results **(E,J)** are NLM correction results. Units: millimeters (1 mm).

These metrics are sensitive to structural and brightness differences caused by lighting changes. The correction images (Figure 14) and corresponding subjective results are given below.

The subjective results indicate that while NLM produces a smoother effect, it also tends to sacrifice more information. This is evident in complex matrix arrangements with repetitive objects. For instance, in Figure 14I, the blood tube at the bottom left remains nearly invisible, demonstrating that under uneven light source distribution (Figure 14), the evaluation metrics influenced by the light source tend to favor algorithms such as ADF. ADF preserves more objects, despite potentially containing more noise and/or artifacts (Figure 14J).

However, the effectiveness of a filter is highly dependent on its parameter settings. The differences in results may be due to the parameter settings of the simulation, which may have been suboptimal for the physical test data. To ensure that the changes in the results of physical verification were not due to the filter parameter design, and to further investigate the effect of uneven light source distribution on artifact removal, we opted for a representative Gaussian light source with a waist radius ranging from 1 to 20 mm, incremented in 1 mm steps. This approach was to simulate the effect of light source distribution on the artifact removal algorithm in cavity structures PAI.

#### 3.2.3 Artifact-removal: Gaussian light source with different waist radii

**FIGURE 15:**
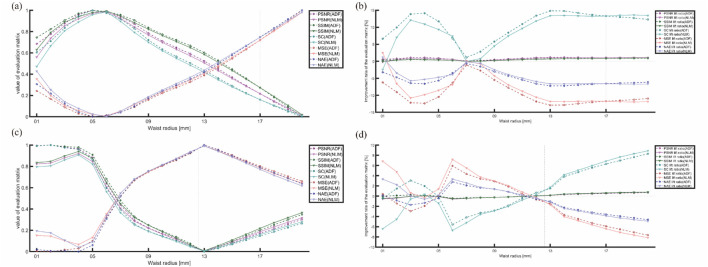
Trends of various metrics with changes in the light source parameter: **(A,C)** trends of normalized metrics changing with the light source variations for the horizontal and matrix arrangements, respectively. **(B,D)** improvement-rate trends for the two algorithms with light source variations for the horizontal (S4) and matrix arrangements (S5).

To address the concerns regarding whether the observed differences between simulation and physical experiments stem from the sensitivity of the objective evaluation metrics to light source variations or from the selection of filter parameters, we visualized the effect of light source parameters on the objective evaluation metrics for both algorithms. This analysis, shown in Figures 15A, C, demonstrates that both algorithms produced improvements in almost all metrics compared with the original image, with each metric undergoing regular changes as the light source varied. By normalizing these metrics, we observed a correlation between the objective evaluation metrics and light source parameter changes. Therefore, the differences between the simulation and the physical experiments were not mainly due to overly sensitive objective evaluation indicators or incorrect filter parameter selection.

Furthermore, multiplicative noise primarily affects the uniformity and consistency of PAI as it is directly proportional to the signal intensity of the image itself. This type of noise presents a particular challenge in PAI because the intensity of the generated signal is directly influenced by the uneven distribution of the light source and variations in its absorption. To mitigate the effect of the light source on these metrics, we focused on the evaluation metrics’ improvement rates for comparing the improvements made by ADF and NLM, as shown in the right-hand columns of Figures 15B, D:

First, in Figure 15, the trends of MSE and MAE are opposite to those of most other metrics. Because these two are negatively correlated with image quality, flipping their trend lines aligns them with the graph of the change in the light source waist radius-quality evaluation metric.

Second, Figures 15A, C show that higher metric values at lower waist radii (3–6 mm) do not necessarily translate to satisfactory improvements. However, under conditions of highly focused (3–5 mm) waist radii in the horizontal arrangement and 6–8 mm in the matrix arrangement or more uniform light sources (12–20 mm) waist radii in the horizontal arrangement and 14–20 mm in the matrix arrangement, the improvement effects are better.

Notably, frequent crossover points in the performance curves of both algorithms at waist radii below 8 mm suggest unreliable data, likely due to excessive focus on the light source, causing decreased attention to the blood tubes (the actual objects of measurement) and excessive attention to visible light paths (incorrectly evaluated objects), rendering this data segment unreliable. However, at waist radii above 10 mm, the improvement situations of both metrics were more uniform and informative for selecting an artifact-removal algorithm.

**FIGURE 16:**
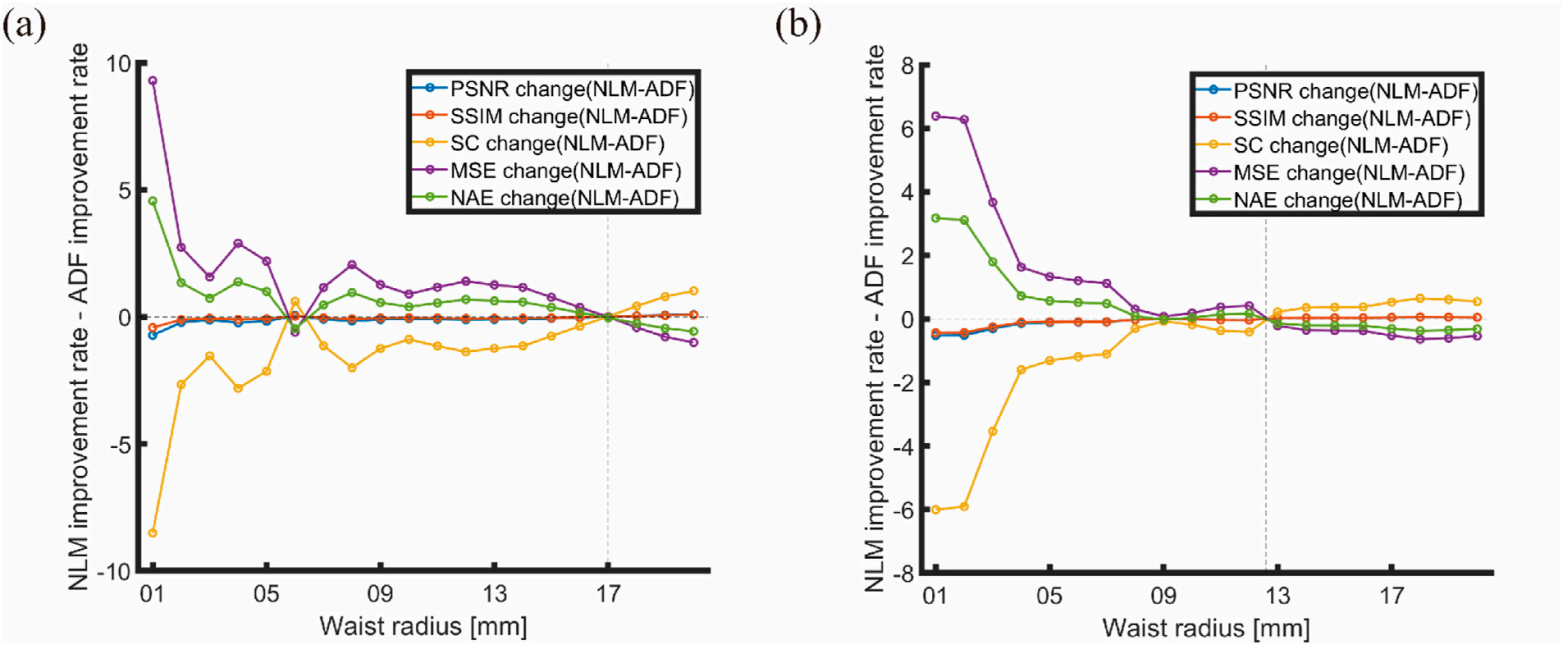
Ratio of ADF to NLM improvement rates against changes in light source: **(A)** Horizontal arrangement (S4); **(B)** Matrix arrangement (S5).

The ratio of ADF to NLM improvement rates against changes in the light source was plotted, as shown in Figure 16.

According to Figure 16, the intersection points of the improvement rate ratios between ADF and NLM occurred at waist radii of approximately 17 and 12.6 mm. For images with a horizontal arrangement (Figure 16A), when the waist radius is above 10 mm and below 17 mm (for matrix arrangement, below 12.6 mm), all positive indicators of improvement are negative, and *vice versa*, indicating that NLM outperforms ADF. Beyond 17 mm, the positive indicators (PSNR and SSIM) are positive, and the negative indicators (MSE and NAE) are negative, indicating that ADF outperforms NLM. This aligns with the simulation and physical verification results for uniform light sources, leading to the conclusion that in our experiments, the distributions of Gaussian light sources, particularly those with waist radii larger than 10 mm, are a primary factor in choosing artifact-removal algorithms for PAI. Analogous considerations apply to other types of light sources based on the Gaussian sources’ distribution scenarios.

### 3.3 Challenges and future perspectives

In these simulation and validation experiments, the tubes were completely cylindrical and arranged horizontally. This setup primarily focused on horizontal layouts because they effectively demonstrate the basic principles of photoacoustic signal generation and artifact propagation and facilitate the control and analysis of interactions among different media (blood, air, water). The shape and angle distribution of actual cavity organs, such as the nasal cavity or the ear canal, will cause different and more challenging artifacts.

As for the Grüneisen parameter, it was not explicitly included in our model, which primarily focuses on simulating photoacoustic wave propagation using acoustic properties obtained through physical experiments. This approach did not specifically consider thermal expansion effects but assumed that the initial sound pressure is proportional to light absorption and the Grüneisen parameter. Consequently, the Grüneisen parameter was set to a unit value of 1, implying that it was not explicitly included in our model configuration. Grüneisen parameter along with additional thermodynamic parameters could be used to further refine the proposed model and enhance the accuracy and applicability of the simulations. By integrating more thermodynamic parameters and refining the physical model, aiming to optimize the photoacoustic simulation framework and explore its potential in biomedical imaging applications.

For image reconstruction, the DAS algorithm was our primary tool to evaluate the effects of artifacts caused by water and air tubes within photoacoustic imaging. Although the DAS algorithm is widely used due to its simplicity and computational efficiency, it lacks optimization for artifact removal to further enhance the quality of image reconstruction and reduce artifacts, other image reconstruction algorithms can be considered while exploring de-artifacting techniques, such as model-driven iterative reconstruction methods and back-projection algorithms. Model-driven iterative reconstruction methods can effectively utilize prior knowledge to optimize the reconstruction process, while back-projection algorithms can improve computational efficiency, potentially offering enhanced flexibility and precision for PAI.

Under conditions of uneven light source distribution, significant shadows were observed when the light source was focused on the air tubes. This currently unavoidable issue can be temporarily mitigated by opting for a more divergent light source to reduce the effect of focused illumination on air.

In practical applications, the optical absorption characteristics and photoacoustic conversion efficiencies of biological tissues may vary due to tissue heterogeneity. To simplify the complexity of the problem and focus on the fundamental relationship between optical absorption and initial sound pressure, this study assumed a uniform medium. While using agarose phantoms has been beneficial for understanding the basic principles and artifact generation in PAI, this approach does not fully capture the heterogeneity of cavity structure tissues, such as the gray and white matter in the brain, and the varying densities of lymph nodes, fluids, and vascular structures in the lungs. Future work will be dedicated to further considering medium heterogeneity, exploring its effect on the generation and propagation of photoacoustic signals, and improving the model to simulate and explain these effects more accurately.

In the complex arrangement conditions typical of PAI, methods such as NLM can be advantageous. NLM is particularly effective for enhancing similar tissue areas, such as continuous vascular structures, while concurrently reducing noise. This characteristic suggests potential benefits for NLM when PAI is applied to complex scenarios, particularly once the problems posed by uneven light source distribution are addressed.

In current image processing research, ADF and NLM algorithms possess unique advantages and limitations. ADF primarily focuses on enhancing the prominent local gradients at edges within images, whereas NLM is aimed at smoothing globally repetitive textures. In practice, these strategies may conflict; for instance, edge enhancement by ADF can interfere with the global smoothing by NLM, resulting in inappropriate blurring near edges, thus negating the advantages of each method.

Particularly, NLM performs poorly under uneven light distribution but excels when the lighting is uniform, ADF is just the opposite. To address this challenge, a potential solution involves integrating a light source distribution detection feature. This feature would assess the light distribution based on specific reference thresholds and combine NLM and ADF appropriately. This approach not only optimizes the application of algorithms to ensure effective image processing under various lighting conditions but also helps resolve the parameter-setting contradictions between ADF and NLM.

Further, future research should develop new integration strategies, potentially in the form of a framework that dynamically selects between ADF and NLM at appropriate processing stages. For example, ADF could be used for edge protection during preprocessing, followed by global smoothing with NLM in the post-processing stage, or a hybrid model could be developed that dynamically selects between ADF and NLM under specific conditions to optimize image quality. This would fully leverage the strengths of both methods and avoid the issues that arise when they are used independently.

This study investigated diffusion methods and filtering algorithms. However, deep learning methods could be investigated but could face a notable challenge: their dependence on large training datasets. Deep learning models, especially CNNs, require extensive data to effectively learn the necessary weights and features. This issue can be addressed using computational models such as k-Wave and synthetic phantoms or even deep reinforcement learning, which allow for the creation of a feedback loop that refines the dataset with high-quality, outcome-focused vast examples. Nevertheless, generating a comprehensive dataset that includes all potential image features likely to be encountered in real-world scenarios remains a significant challenge and an area ripe for further research.

## 4 Conclusion

### 4.1 Mechanism of artifact generation

Artifacts in acoustic imaging can primarily be attributed to variations in sound velocity and differences in acoustic impedance within a medium. Variations in sound velocity affect the resolution of the reconstructed image, whereas mismatched acoustic impedances cause signal reflections, forming artifacts. In anisotropic image reconstructions, particularly around blood vessels, semicircular arc-shaped artifacts caused by air ducts are noticeable. By contrast, such semicircular arc-shaped artifacts are absent in image reconstructions using isotropic acoustic models. This may be due to the more uniform propagation of sound waves in isotropic models, which reduce the strong reflections caused by impedance mismatches, not having these semicircular arc-shaped artifacts. This observation suggests the significant effect of acoustic impedance mismatches on artifact formation, especially in complex biological tissues. Minimizing this impedance mismatch is therefore crucial for improving acoustic imaging quality.

### 4.2 Shapes of artifacts

Larger distances between the blood and air tubes cause the artifacts surrounding the blood tube to have larger diameters. Meanwhile, the presence of the water tube does not produce a greater number of artifacts compared to the gas tube because blood and water have similar acoustic properties, although the water tube is more absorptive than gas tube. The use of a half-ring array of ultrasound sensors further contributes to the semicircular shape of these artifacts.

### 4.3 Interaction between the imaging of blood vessels

In the simulation with a matrix arrangement, the imaging of the vessel changes from a point in the original simple object arrangement to an arc. This may be owing to the mutual influence when imaging multiple vessels, as the semicircular arc imaging of the vessel does not appear in the pure artifact image. Otherwise expressed, the mutual influence during the imaging of multiple vessels without the effect of air ducts can also change the imaging of the vessel from a “point” to an arc.

### 4.4 Performance of filtering algorithm

Regarding the performance of filtering algorithms, NLM outperforms ADF in simulations with a uniform light source. Conversely, in physical verifications with non-uniform light sources, NLM underperforms compared to ADF. The intersection points of the ratio between the improvement rates of the evaluation metrics for ADF and NLM mostly occur at waist radii of 17 and 13 mm (for both the simple and matrix arrangements). While the metrics we selected are sensitive to changes in the light source parameter, each metric exhibited a consistent pattern of changes. In addition, when the waist radius of the Gaussian light source exceeds 10 mm, the evaluation metrics for the optimization rates of the algorithms tend to stabilize, providing clearer guidance for choosing the appropriate artifact-removal algorithm. In environments with uniform light fields, NLM leverages uniform environmental areas for more effective filtering.

### 4.5 Future considerations for artifact removal using objective evaluation metrics

To evaluate the differences between the two algorithms, appropriate experimental assessment metrics must be selected. Nevertheless, the discrepancies between objective evaluations and subjective perceptions pose challenges. While the five chosen objective image quality assessment indicators typically correlate with subjective evaluations for Gaussian radii above 10 mm, an anomaly occurs for Gaussian light source waist radii below 10 mm, characterized by peaks in parameters but valleys in improvement rates, complicating the explanation. When grid searches for light source-filter parameters are conducted in future image training processing or enhancement algorithms, ensuring a sufficiently large light-source waist radius is critical to avoid such anomalies and ensure accurate and effective artifact removal.

## Data Availability

The raw data supporting the conclusions of this article will be made available by the authors, without undue reservation.
